# Lactate Dehydrogenase Gene Family in *Spirometra mansoni* (Cestoda: Diphyllobothriidea)—Phylogenetic Patterns and Molecular Characteristics

**DOI:** 10.3390/ani13233642

**Published:** 2023-11-24

**Authors:** Shasha Liu, Ke Zhou, Fei Gao, Wen Li, Zhongquan Wang, Xi Zhang

**Affiliations:** Department of Parasitology, School of Basic Medical Sciences, Zhengzhou University, Zhengzhou 450001, China; qwelss202107@163.com (S.L.); zzhouxke@163.com (K.Z.); gaofei199812@163.com (F.G.); liwen01232022@163.com (W.L.); wangzq@zzu.edu.cn (Z.W.)

**Keywords:** cestode, lactate dehydrogenase, identification, phylogeny, enzymatic traits

## Abstract

**Simple Summary:**

*Spirometra mansoni* is a neglected human-infective tapeworm. Its motile larva can invade humans, causing a parasitic zoonosis known as sparganosis. Cestoda parasites live in an oxygen-deprived environment in the gastrointestinal tract of their hosts; they mainly depend on energy production from glucose to lactate catalyzed by a series of enzymes under anaerobic conditions. Meanwhile, lactate dehydrogenase (LDH) is a complicated multi-gene family and plays key roles in oxidative production capacity and detoxification pathways in organisms. The purpose of this study was to investigate the gene diversity, phylogenetic patterns and molecular characteristics of the *S. mansoni* lactate dehydrogenase (*Sm*LDH) protein family to explore the potential important roles of *Sm*LDH in the growth and development of *S. mansoni*.

**Abstract:**

The plerocercoid of *Spirometra mansoni* can parasitize both human and animals, resulting in sparganosis. Lactate dehydrogenase (LDH) is an important enzyme in parasites. However, our knowledge of the LDH family in *S. mansoni* is still inadequate. This work identified 19 new LDH members in *S. mansoni*. Clustering analysis demonstrated that all *Sm*LDHs were divided into two main groups, which is consistent with the patterns of conserved motif organization. According to RT-qPCR, 2 LDHs were highly expressed in the plerocercoid stage and 17 LDHs were highly expressed in the adult stage. The evolutionary tree showed a high level of diversity of both cestode and trematode LDHs. *Sm*LDHs contained both conserved family members and members in the process of further diversification. r*Sm*LDH has a NAD-binding domain and a substrate-binding domain. The protein was immunolocalized in the epidermis of the pleroceroid and in the tegument, uterus and egg shell of adult worms. The optimum activity for r*Sm*LDH in the pyruvate reduction reaction was found to be pH 4.5 and 37 °C. In the oxidation reaction, optimal values for pH and temperature were 9.0 and 30 °C, respectively. Gossypol was found to be the most powerful inhibitor in both reduction and oxidation reactions. The results provide a basis for the further study of the biological roles of LDHs in *S. mansoni* and other LDH-containing taxa.

## 1. Introduction

The motile larva (plerocercoid) of the tapeworm *Spirometra mansoni* can invade humans, causing a parasitic zoonosis known as sparganosis [1]. More than 2000 human sparganosis cases have been reported worldwide so far, with the majority coming from Eastern and Southeastern Asia [2]. The majority of human infections result from eating undercooked meat from frogs or snakes infected with plerocercoids, or through drinking untreated water containing *Spirometra* larvae [3,4]. Although it is of medical importance, *S. mansoni* has been neglected for a long period of time, and our knowledge about the molecular interaction between the parasite and its host is still fragmentary [5,6]. Therefore, addressing this knowledge gap is an urgent matter.

Lactate dehydrogenase (LDH) is a complicated multi-gene family and a widely distributed biocatalyst that plays key roles in oxidative production capacity and detoxification pathways in organisms [7]. The LDH family can simply divide into two sub-families, L-LDH and D-LDH, according to different conformations of lactic acid generated by each sub-family. The two sub-families are not evolutionarily related, with D-LDH belonging to the D-configuration 2-hydroxyacid dehydrogenase and L-LDH belonging to the L-configuration of NAD+-dependent dehydrogenase [8]. Both L-LDHs and D-LDHs contain the NAD+ binding site sequence GXGXXGX (where X is any one of 20 amino acids) and two structural domains: the coenzyme-binding structural domain and the substrate-binding structural domain [9]. Metazoans only contain the type L-LDH, which is a key enzyme in the process of energy metabolism.

LDH catalyzes the reversible reaction between pyruvate and lactate with the aid of reduced nicotinamide adenine dinucleotide (NADH) and oxidized nicotinamide adenine dinucleotide (NAD); once inhibited, it will lead to a cessation of organism development or even death [10]. Helminth parasites live in the digestive tract within the an oxygen-deprived environment of their hosts, and they mainly depend on energy production from glucose to lactate, catalyzed by a series of enzymes under anaerobic conditions [11]. In addition, LDHs have been proven to be good diagnostic molecules and potential anthelminthic drug targets or vaccines [12]. Owing to the important functions and roles of LDH, a series of studies have been conducted on this enzyme in different tapeworms [13,14].

Previous comparative genomic and transcriptomic studies revealed that LDH is a multi-copy and high-expression family in the *Spirometra* tapeworm, suggesting potential important roles of LDH in the adaptive parasitism of *Spirometra* tapeworms [15,16]. However, little is known about the family’s polymorphism and the molecular characteristics of LDH in *S. mansoni*. Therefore, in this study, we first investigated the diversity of protein family members in *S. mansoni* as well as phylogenetic patterns of LDHs in platyhelminthes; then, the molecular characteristics of lactate dehydrogenase were further explored.

## 2. Materials and Methods

### 2.1. Ethical Approval

The study was conducted in accordance with the guidelines of the Declaration of Helsinki, and all procedures involving animals were approved by the Life Science Ethics Committee of Zhengzhou University (permit code SYXK 2021-7023). The animals were handled in accordance with good animal practices, as required by the Animal Ethics Procedures and Guidelines of the People’s Republic of China.

### 2.2. Experimental Animals

The plerocercoids were initially isolated from wild frogs and identified as *S. mansoni* via molecular typing based on the method detailed in [3]. Then, plerocercoids were maintained in our lab via serial passage in mice every 10–12 months [17]. Fifteen female BALB/c mice (6-week-old) were used for immunization by antigens of recombinant lactate dehydrogenase (rLDH) for 4 times to obtain the anti-r*Sm*LDH serum [18]. The interval between immunizations was two weeks for two months in total. Collected anti-r*Sm*LDH serum was stored at −80 °C until use. An adult worm of *S. mansoni* was obtained from an infected cat as described previously [19]. Proglottids collected from the adult were used for the subsequent experiments.

### 2.3. Identification of SmLDH Family Members

NCBI-CDD “www.ncbi.nlm.nih.gov/Structure/cdd/wrpsb.cgi” (accessed on 1 January 2023) was used to find genes encoding structural domains containing LDH proteins. The obtained sequence was preliminatively identified as lactate dehydrogenase using a reference sequence of Pfam (PF00582). All candidates were further analyzed using HMMER “https://www.ebi.ac.uk/Tools/hmmer/” (accessed on 4 January 2023) to confirm the presence of key domains. Gene structure Display Server (GSDS) v2.0 “http://gsds.gao-lab.org/index.php” (accessed on 2 January 2023) was used to analyze the gene characteristics of *Sm*LDHs. The multi-sequence alignment was performed in DNAMAN v9.0 (Lynnon BioSoft, Quebec, QC, Canada). Using the LG + G model, the phylogenetic tree was generated through the maximum likelihood (ML) approach in MEGA v7 [20]. LDH protein structures were predicted using the PSIPRED service “http://bioinf.cs.ucl.ac.uk/psipred/” (accessed on 24 January 2023). Utilizing homology modeling, which is available on the SwissModel service “https://swissmodel.expasy.org/” (accessed on 24 January 2023) the three-dimensional structure was established. Swiss-PdbViewer v.4.1 was used to show the results of the Ramachandran plot analysis [21].

### 2.4. Quantitative RT-PCR Analysis

Based on gene-specific primers, quantitative RT-PCR (qRT-PCR) analysis was utilized to track *Sm*LDH expression levels in the plerocercoid stage as well as in several proglottides (immature proglottide, mature proglottide, and gravid proglottide) of the adult stage (Supplementary Table S1). A 7500 Fast Real-time PCR machine (Applied Biosystem, Monza, Italy) was used to perform qRT-PCR. The reaction mixture contained 10 µL of 2 × TB Green Premix Ex Taq (Takara, Beijing, China), 10 μM each of sense and antisense primer, and100 ng of first-strand cDNA. Initial thermal cycling at 95 °C for 30 s followed by 40 cycles of 95 °C for 3 s and 60 °C for 30 s was conducted. GAPDH gene was used as an internal control [5]. The comparative 2-ΔΔCT approach was used to analyze relative gene expression levels [22].

### 2.5. Phylogenetic Analysis

All available LDH sequences of medical cestodes and trematodes were also extracted from the WormBase ParaSite database. The mixture model was was used to analyze potential motifs using the expectation maximization (MEME) method [23]. Using MAFFT v7, multiple-protein sequence alignment was carried out [24]. Bayesian inference (BI) and maximum likelihood (ML) were used to conduct phylogenetic analysis. BI analysis was performed in MrBayes v.3.2 [25]. The analysis consisted of two runs, each with four MCMC chains running for 5,000,000 generations, and sampling at every 100th generation. ML analysis was performed in MEGA v.7.0 [20]. Confidence in each node was assessed via boot-strapping (1000 pseudo-replicates).

### 2.6. Molecular Identification of rSmLDH

The *Sm*LDH1 gene was amplified via PCR with specific primers carrying BamHI and PstI restriction enzyme sites (forward, 5′-AT**GGATCC**ATGCCACAAACTATG-3′; reverse, 5′-TA**AAGCTT**TCACCAGTTGACACCG-3′). The final PCR products were purified, digested, and cloned into the pQE-80L vector (Ipswich, Essex County, MA, USA). Recombinant plasmid was then transformed into *Escherichia coli* BL21. The expression of r*Sm*LDH was induced by adding 0.1 mM IPTG at 33 °C overnight. SDS-PAGE was used to identify r*Sm*LDH1. Using an indirect ELISA, antibody titrations from immunized mice were found. Each well of 96-well plates was coated with r*Sm*LDH protein overnight at 4 °C before being blocked with Tween 20 (PBST) containing 5% skim milk at 37 °C for two hours. Each well received 100 μL of diluted immune serum, which was then incubated at 37 °C for two hours. Goat anti-mouse IgG (HRP-labeled) was added in a dilution of 1:5000 and incubated for 1 h at 37 °C. After 15 min of incubation, 100 μL of OPD chromogen substrate containing H_2_O_2_ was added to each well, and the reaction was terminated by adding 50 μL of 2 M H_2_SO_4_. A computer-controlled BioTek (Synergy LX, WINOOSKI Contry, VT, USA) microplate reader was used to measure the optical density (OD) of each well at 490 nm. The location of *Sm*LDHs was determined using an indirect immunofluorescence assay (IFA). After retrieving the tissue samples from the plerocercoids and adult worms, they were blocked with 5% normal goat serum in PBS and then incubated at 37 °C for 60 min with a 1:10 dilution of anti-r*Sm*LDH serum, mouse serum from plerocercoid-infected mice, normal mouse serum, or PBS. Propidium iodide (PI) was used to stain the nuclei after the sections were treated with a 1:50 dilution of FITC-labeled anti-mouse IgG (Santa Cruz, CA, USA) at 37 °C for 15 min. The slices were finally viewed under a fluorescence microscope (Olympus, Japan) after 5 PBS washes.

### 2.7. Enzyme Activity Assays

In the reduction reaction, 0.5 mM NADH and 10 mM pyruvate were used as substrates to determine enzyme activity in 100 mMTris-HCL (pH 9), and incubated at 37 °C for 5 min. In the oxidation reaction, 1 mM NAD and 50 mM lactate were used as substrates to determine enzyme activity in 100 mMTris-HCL (pH 4.5), and incubated at 33 °C for 5 min. The effect of different factors (temperatures, pH, substrates and cofactors) on r*Sm*LDH was determined by keeping the enzyme in different conditions for 5 min, and then enzyme activity was assayed immediately. The absorbance changes were observed at 340 nm (ε ¼ 9.6 mM^−1^·cm^−1^). The apparent *Km* and *Vmax* values in the kinetic mechanism study were graphically determined using the double-reciprocal Lineweaver–Burk plot. Studies on inhibition were carried out using the method of Chen et al. [5]. The typical reaction system was supplemented with the inhibitors (thionicotinamide, nicotinic acid, gossypol, and oxalate) that would be put to the test. Plotting the percent of residual activity vs. the log of inhibitor concentration yielded the concentration of the inhibitor producing 50% inhibition (IC50). Methods described in Nava et al. [26] were used to determine the *Ki* values for each r*Sm*LDH inhibitor. Initial-velocity enzyme reactions with the inhibitors were carried out to identify the type of reversible enzyme inhibition. In addition, the inhibitory effect of the anti-r*Sm*LDH antibody on the r*Sm*LDH enzyme was determined. r*Sm*LDH (10 μg) was incubated with immune serum and blank serum for 1 h at 37 °C with serum concentrations of 4.8, 2.4, 1.2, 0.6 and 0.3 μg. Relative enzyme activity was defined as the enzyme activity of the antibody incubation group divided by the enzyme activity of the blank serum group at the corresponding concentration.

## 3. Results

### 3.1. The Lactate Dehydrogenase Gene Family in S. mansoni

**Figure 1:**
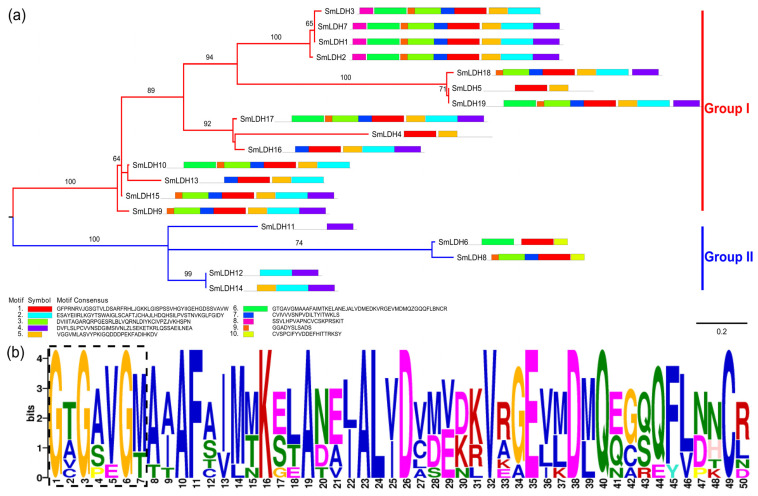
Lactate dehydrogenase (LDH) sequences in *Spirometra mansoni*. (**a**) Cluster analysis and conserved motifs of 19 *Sm*LDHs. Numbers on the branches are self-extending values; only values above 60 are shown. (**b**) NAD+-binding motif (G-X-G-2X-G-X).

**Figure 2:**
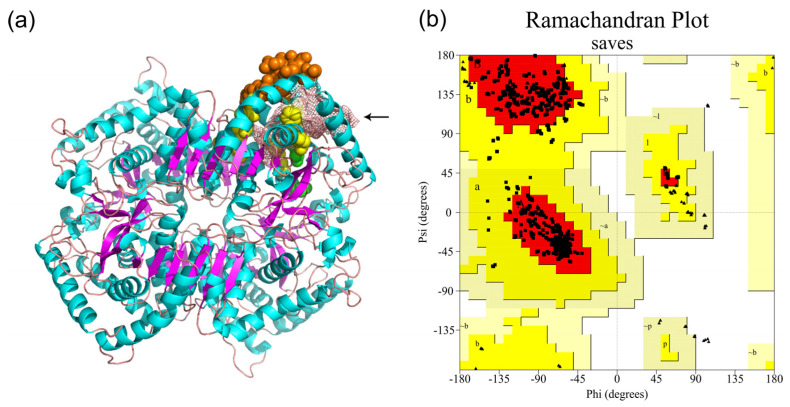
Homology modeling and model quality evaluation of lactate dehydrogenase in *Spirometra mansoni*. (**a**) Predicted three−dimensional structure of *Sm*LDH. The α−helix, β−sheet layer and loop ring are blue, purple and pink, respectively. The orange, yellow and green spheres represent the 104 aa−111 aa region, NAD+−binding sites and pyruvate−binding sites, respectively. “←” represents the catalytic site of the pink reticulum. (**b**) Red indicates the optimal region; brown indicates the subpermissive region; yellow indicates the general permissive region; and the light color represents the impermissive region.

**Figure 3:**
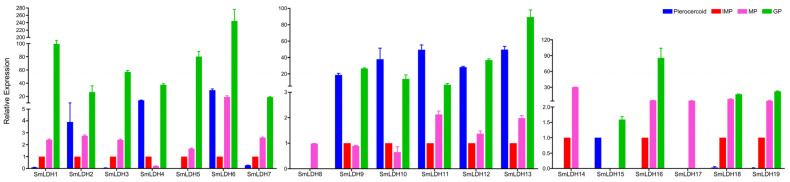
The mRNA levels of the *Sm*LDH gene in different tissues of *Spirometra mansoni*. Quantitative analysis was performed to determine mRNA levels of lactate dehydrogenase encoding *S. mansoni* in the plerocercoid, and immature, mature and gravid proglottid. The expression level was normalized to GAPDH and measured with the 2^−ΔΔCt^ value. Results are averaged from three independent replicates during all stages. Error bars represent the SD (n = 3). Immature proglottids were selected as controls (taking a value of 1) and mRNA levels were calculated for plerocercoid, mature proglottid and gravid proglottid relative to immature proglottid expression. IMP, immature proglottide; MP, mature proglottide; GP, gravid proglottide.

In total, 19 LDH members were identified in *S. mansoni*. The length of *Sm*LDHs ranged from 282 bp to 3021 bp, and the predicted protein length ranged from 93 aa to 367 aa (Table 1). Clustering analysis revealed that the *Sm*LDHs can be arranged into two groups: Group I and Group II. Group I contained 14 SmLDHs, and Group II had 5 members (Figure 1a). The functional motif “IGEHGDS” was present in most *Sm*LDHs. In addition, there was a NAD+-binding motif (G-X-G-2X-G-X) distributed in eight *Sm*LDHs (LDH1, LDH2, LDH3, LDH6, LDH7, LDH10, LDH17, and LDH19) (Figure 1b). According to the modeled 3D structure of *Sm*LDH, both NAD-binding residues (yellow) and pyruvate-binding residues (green) were located on α-helix and loop rings. The epitopes 104 aa–111 aa formed a ring on the protein surface. The key catalytic sites 112 R, 172 D and 199 H formed a catalytic center that is connected to the vicinity of the ring (Figure 2a). The Ramachandran plot showed that more than 98% of the amino acid residues were in the allowed region, indicating the good quality of the protein model (Figure 2b). In total, 15 genes were found to be expressed in both the plerocercoid and adult stages. Among these, 12 genes were highly expressed in the adult stage, while in the plerocercoid stage, 2 genes (LDH10 and LDH11) were highly expressed. In the adult stage, in total, 16 expressed genes were detected, and 13 genes were highly expressed in the gravid proglottid, while LDH8 and LDH17 were only expressed in the mature proglottid (Figure 3).

### 3.2. LDHs in Medical Platyhelminthes

**Figure 4:**
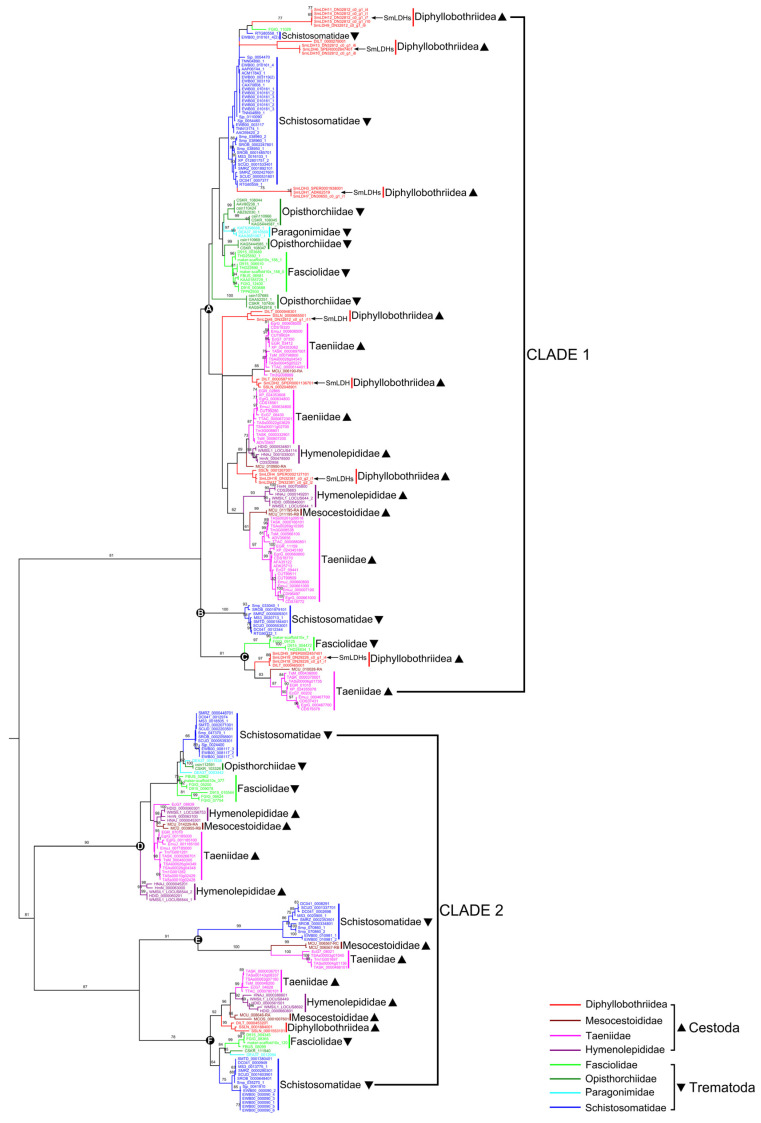
Phylogenetic analysis of lactate dehydrogenase sequences of medical cestodes and trematodes based on the maximum likelihood method. The numbers on the branches represent bootstrap values, and only values with bootstrap values greater than 60 are displayed.

In total, 273 LDH sequences in 15 cestodes and 14 trematodes were retrieved (Supplementary Table S2). The combined dataset of cestode and trematode was analyzed using both ML and BI methods. The tree topologies obtained were consistent. Here, we only use the ML tree to show the phylogenetic relationships of LDHs among medical platyhelminthes. Although two main branches, CLADE 1 and CLADE 2, were revealed, no groups were clustered according to their taxonomic systems, and species in all cestodes and trematodes were dispersed in each generated clade (Figure 4). Specifically, CLADE 1 contained three main groups: Groups A, B and C. Most of the samples were collected in Group A, including species from Diphyllobothriidea, Taeniidae, Hymenolepididae and Mesocestoididae of cestodes, as well as samples from Fasciolidae, Opisthorchiidae, Paragonimidae and Schistosomatidae of trematodes. Group B was a monophyletic group containing only the LDH of Schistosomatidae; Group C contained sequences of Fasciolidae, Diphyllobothriidea, Taeniidae and a Mesocestoididae. CLADE 2 consisted of two subclades that were sister groups. The first subclade was composed of the monophyletic group, Group D, and the other was composed of two groups: Group E and Group F. For *S. mansoni*, its members were mainly distributed in Group A, and only three sequences were inserted into the Group C. However, *Sm*LDHs were not clustered in Group A, but dispersed in six different branches.

### 3.3. Molecular Characterization of rSmLDH

**Figure 5:**
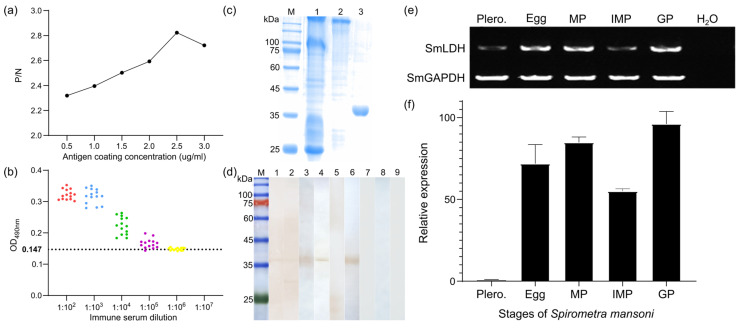
Molecular characterization of cloned *Sm*LDH. (**a**) Determination of optimal antigen encapsulation concentration; (**b**) anti-r*Sm*LDH immunoserum potency assay. Red, blue, green, purple, yellow, and cyan represent serum dilutions of 1:10^2^, 1:10^3^, 1:10^4^, 1:10^5^, 1:10^6^, and 1:10^7^, respectively; (**c**) SDS-PAGE analysis of plerocercoid soluble antigen, ES antigen and purified protein. M: protein pre-stained marker; Lane 1: plerocercoid soluble antigen; Lane 2: plerocercoid ES antigen; Lane 3: r*Sm*LDH purified protein; (**d**) plerocercoid antigenicity analysis. M: protein pre-stained marker; Lanes 1, 4, and 7: plerocercoid soluble antigen; Lanes 2, 5, and 8: plerocercoid ES antigen; Lanes 3, 6, and 9: r*Sm*LDH purified protein; Lanes 1–3: serum identification by anti-r*Sm*LDH mice; Lanes 4–6: serum identification by plerocercoid-infected mice; Lanes 7–9: serum identification by normal mice. (**e**,**f**) The transcription pattern of the LDH gene in different developmental stages of *Spirometra mansoni*. Conventional RT-PCR (**e**) and real-time RT-PCR (**f**) performed on cDNA from various developmental stages of *S. mansoni*, including eggs, plerocercoids and adults. A house keeping gene (GAPDH) was used as a positive control. H_2_O was used as a negative control. Plero.: plerocercoid; MP: mature proglottid; IMP: immature proglottid; GP: gravid proglottid.

**Figure 6:**
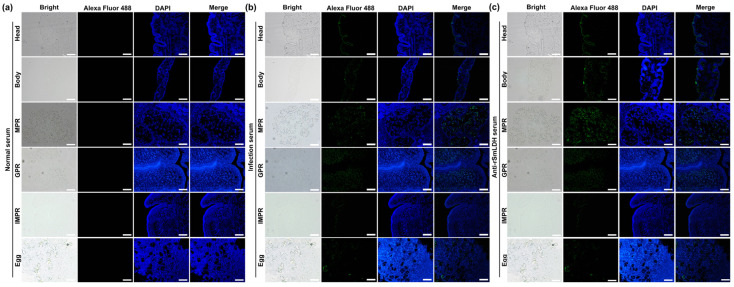
Immunofluorescence localization of LDH in different developmental stages of *Spirometra mansoni*. Head: head of proglottid; Body: body of plerocercoid; MPR: mature proglottid; GPR: gravid proglottid; IPR: immature proglottid; Egg: eggs in the uterus of gravid proglottid. (**a**) Normal serum; (**b**) infection serum; (**c**) anti-*Sm*LDH serum. Scale of different segments of adult: 500 μm; head of plerocercoid: 200 μm; body of plerocercoid: 500 μm; egg: 100 μm.

*Sm*LDH1 is a cellular protein with a predicted Mw of 36.03 kDa and a pI of 6.38 that has a NAD-binding domain and a substrate-binding domain (Supplementary Figure S1). Using the prokaryotic expression vector pQE-80L, *Sm*LDH1 was cloned. PQE-80L-*Sm*LDH1, a recombinant plasmid, expressed a soluble fusion protein. In the meantime, r*Sm*LDH protein-based indirect ELISA was developed. The ideal conditions were 2.5 g/mL of protein and a 1:100 dilution of mouse serum (Figure 5a). The cut-off value of 0.147 served as the benchmark for the tests that followed (Figure 5b). According to SDS-PAGE, r*Sm*LDH had a molecular weight of about 36 kDa (Figure 5c). An amount of 1.5 mg/mL of r*Sm*LDH was present in the sample. Western blotting showed that r*Sm*LDH, an excretory–secretory antigen, and a soluble antigen of plerocercoid could be recognized by the anti-r*Sm*LDH serum. Serum from infected mice identified the soluble plerocercoid antigen and r*Sm*LDH with high specificity (Figure 5d). *Sm*LDH gene mRNA transcription was seen in all proglottid stages, as well as the plerocercoid and egg stages. (Figure 5e). A qPCR study revealed that the gravid proglottid stage had the highest transcriptional level, followed by the mature proglottid stage and the egg stage (Figure 5f). The immunolocalization test revealed that utilizing anti-r*Sm*LDH serum, particular fluorescent staining was seen in egg shells in the uterus. Subcutaneous fluorescence staining was seen in the plerocercoid stage. In the adult stage, fluorescence staining was observed in the tegument of each proglottid, as well as in the parenchyma (Figure 6).

### 3.4. Enzyme Kinetics and Inhibition Studies

**Figure 7:**
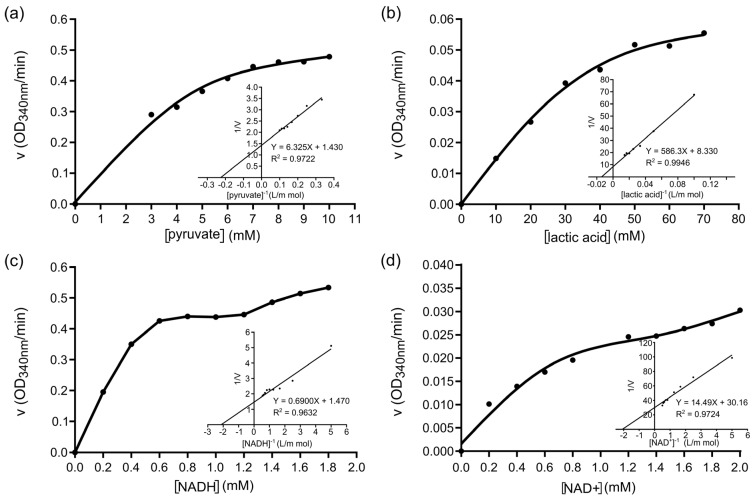
Substrate concentration effect on r*Sm*LDH enzyme activity. (**a**) Effects of the substrate concentration of pyruvate on r*Sm*LDH enzymatic activity. The kinetic parameters, *Km* and *Vmax*, were determined using the Lineweaver−Burk plot. The *Km* and *Vmax* values were 4.43 mM and 1.876 μmol min^−1^ mL^−1^, respectively. (**b**) Effects of the substrate concentration of lactic acid on r*Sm*LDH enzymatic activity. The *Km* and *Vmax* values were 75.19 mM and 0.338 μmol min^−1^ mL^−1^, respectively. (**c**) Effects of the substrate concentration of NADH on r*Sm*LDH enzymatic activity. The *Km* and *Vmax* values were 0.49 mM and 1.795 μmol min^−1^ mL^−1^, respectively. (**d**) Effects of the substrate concentration of NAD+ on r*Sm*LDH enzymatic activity. The *Km* and *Vmax* values were 0.48 mM and 0.088 μmol min^−1^ mL^−1^, respectively.

The affinity-purified r*Sm*LDH showed that the optimum temperature for enzyme activity in catalyzing the reduction of pyruvate to lactate was 37 °C, while the optimum temperature in catalyzing the oxidation of lactate to pyruvate was 30 °C. The enzyme activity varied with changes in the pH value. The maximum LDH activity in catalyzing the reduction and oxidation was observed at pH 4.5 and pH 9.0, respectively (Supplementary Figure S2). The calculation of the maximum rate of the recombinant protein-catalyzed reaction of the Michaelis constant (*Km*), reaction rate (*Vm*) and conversion rate (*Kcat*) under different concentrations of pyruvate, NADH, lactate and NAD+ showed that the concentration and reaction rate of pyruvate and lactic acid were plotted as rectangular hyperbolic curves. Initially, the reaction rate increased along with the increase in substrate concentration under low substrate concentrations; then, the rise in the reaction rate decreased gradually as the substrate concentration increased (Figure 7a,b). In addition, the concentration of coenzymes NADH and NAD+ were plotted against the reaction rate revealed in an “S” curve. In the presence of the NADH coenzyme, r*Sm*LDH catalyzes the reduction of pyruvate at a higher rate than that of the oxidation of lactate in the presence of NAD+ (Figure 7c,d).

**Figure 8:**
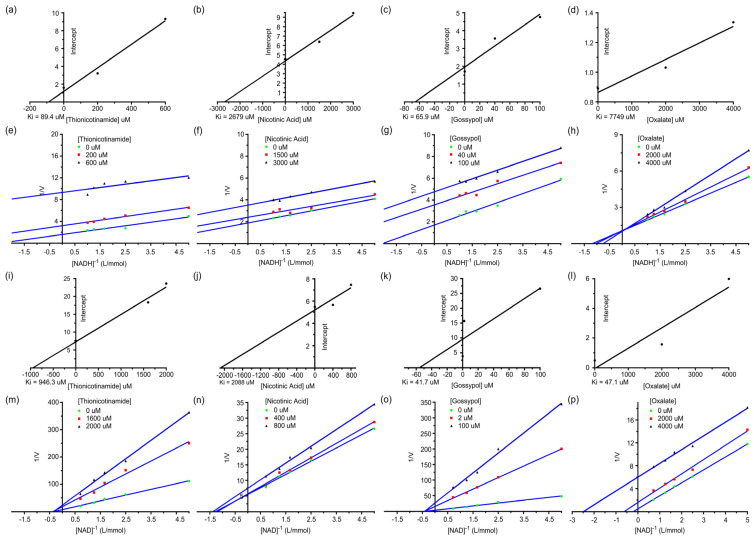
Different inhibitors’ effect on r*Sm*LDH enzyme activity. (**a**–**h**) show the pyruvate reduction of r*Sm*LDH: (**a**) the effect of different concentrations of Thionicotinamide (Thio) on the initial velocities; (**b**) the effect of different concentrations of Nicotinic Acid (Niacin) on the initial velocities; (**c**) the effect of different concentrations of Gossypol (Gsp) on the initial velocities; (**d**) the effect of different concentrations of Oxalate on the initial velocities. The inset shows a secondary plot of the 1/*Vmax* values derived from the primary Lineweaver–Burk plot vs. concentration for the determination of *Ki*. (**e**) Thio (200, 600 μM); (**f**) Niacin (1500, 3000 μM); (**g**) Gsp (40, 100 μM); (**h**) Oxalate (2000, 4000 μM). (**i**−**p**) show the lactate oxidation of r*Sm*LDH: (**i**) the effect of different concentrations of Thionicotinamide (Thio) on the initial velocities; (**j**) the effect of different concentrations of Nicotinic Acid (Niacin) on the initial velocities; (**k**) the effect of different concentrations of Gossypol (Gsp) on the initial velocities; (**l**) the effect of different concentrations of Oxalate on the initial velocities. The inset shows the secondary plot of the 1/*Vmax* values derived from the primary Lineweaver–Burk plot vs. concentration for the determination of *Ki*. (**m**) Thio (1600, 2000 μM); (**n**) Niacin (400, 800 μM); (**o**) Gsp (2, 100 μM); (**p**) Oxalate (2000, 4000 μM).

In the inhibition studies, IC50 results for the r*Sm*LDH were dependent on the inhibitor used (Supplementary Figure S3). Among Thionicotinamide, Nicotinic Acid, Gossypol and Oxalate, the Gossypol was the most powerful inhibitor in both reduction and oxidation reactions, with IC50 values of 86.68 and 2.407 × 10^−12^ μM, respectively. The plotting of the 1/v (μM min^−1^) axis intercept against the inhibitor concentration yielded *Ki* values. In the reduction reaction, the *Ki* values of Thionicotinamide, Nicotinic Acid, Gossypol and Oxalate were 89.4, 2679, 65.9 and 7749 μM, respectively (Figure 8a–d). Thionicotinamide, Nicotinic Acid, and Gossypol were anti-competitive inhibitors, while Oxalate was a competitive inhibitor (Figure 8e–h). In the oxidation reaction, the *Ki* values were 946.3, 2088, 41.7 and 47.1 μM (Figure 8i–l). Thionicotinamide, Nicotinic Acid, and Gossypol were noncompetitive inhibitors in the oxidation reaction, while Oxalate was an anti-competitive inhibitor (Figure 8m–p). The inhibitory effect of anti-r*Sm*LDH antibodies on r*Sm*LDH was also tested. The results showed that the enzyme activity of r*Sm*LDH was mildly inhibited in the pyruvate reduction reaction at an antibody dilution of 1:1000, while it was promoted in the lactate oxidation reaction. At dilutions of 1:500–1:50, the antibody produced a dose-dependent promotion of enzyme activity in the pyruvate reduction reaction and a dose-dependent inhibition of enzyme activity in the lactate oxidation reaction (Supplementary Figure S4).

## 4. Discussion

Lactate dehydrogenase, the terminal enzyme of anaerobic glycolysis, catalyzes the conversion of pyruvate into lactate and plays an essential role in anaerobic conditions when glycolysis is the only way to provide energy [27]. In this study, based on currently known databases, we identified 19 *Sm*LDH family members in total. The analysis of conserved motifs showed that eight members of the family contain the NAD+-binding motif (G-X-G-2X-G-X). A further analysis of its structure revealed that LDH is dominated by the α-helix and irregularly coiled structures. The function of LDH is determined by its spatial structure; α-helix plays a stabilizing role for the backbone, while a β-turn and irregular curl often constitute the enzyme active site and other protein-specific functional sites [13]. Quantitative analysis showed two genes highly expressed in the plerocercoid stage and 17 genes highly expressed in the adult stage, suggesting that LDH genes probably have important roles in the adult stage.

In order to obtain the recombinant protein, we used the *E. coli* expression system, which has various advantages such as simple operation, a short cycle time, low costs and a high yield [28]. However, when using a prokaryotic expression system to express recombinant proteins, different induction conditions have significant effects on the amount of protein expression [29]. Therefore, the conditions for inducing protein expression were continuously optimized. The induced expression of proteins under optimal conditions was present in both supernatant and inclusion bodies. Considering that the purification steps of proteins present in inclusion bodies are complicated and the proportion of active proteins obtained is low, we purified the supernatant lysate protein for subsequent protein functional studies. SDS-PAGE results showed a single target band and few spurious bands, indicating a good purification effect. ELISA results showed high serum antibody potency levels. Western blotting showed that the immunized sera specifically recognized the recombinant antigen, plerocercoid ES antigen and plerocercoid soluble antigen, indicating that the antigen was present in both plerocercoid and the excretion. In addition, the plerocercoid-infected sera recognized recombinant antigen and plerocercoid-soluble antigen, indicating that plerocercoids can stimulate the immune response in mice. The expression of different types of LDH genes varied in different species and tissues. *Babesia* LDH was localized on the cell membrane of infected erythrocytes [30]. Gan et al. [14]. expressed four antigenic epitopes of *Echinococcus granulosus* LDH (*Eg*LDH) and obtained antibodies against each epitope. The immunofluorescence localization of antigenic epitopes was performed on the protoscolex of *E. granulosus* using the above antibodies, and the results showed that strong fluorescence could be observed on the surface of the protoscolex peritoneum. *Taenia asiatica* LDH was located in the superficial membrane of the adult and the germinal membrane of the egg [31]. Here, *Sm*LDHs were detected as expressed in the egg, plerocercoid and adult stages, and the highest expression was in the adult stage. Indirect immunofluorescence experiments showed that the LDH of the plerocercoid stage is mainly present in the cortex, and adult LDH is mainly present in the eggshell in the uterus. Both immune serum and infected serum had weak recognition signals for antigens, which is speculated to be related to the “cold degeneration” characteristic of LDH; that is, the structure of LDH protein will be destroyed at a low temperature, reducing or even losing its antigenicity, and therefore, specimens need to be kept at a low temperature during preparation. We used purified protein for enzyme activity experiments. SDS-PAGE showed a single and clear band at the target position. Using this purified protein for enzyme activity experiments can exclude the influence of heterogeneous proteins on the experiments as much as possible. LDH enzymatic activity reactions are reversible. The rate of pyruvate reduction catalyzed by rLDH under the NADH coenzyme was much higher than that of lactate oxidation catalyzed by NAD+. The *Kcat* value can be used to compare the conversion rate of the reaction [32]. Comparing the *Kcat* values between pyruvate and lactate showed that the conversion rate of the former was higher than that of the latter, while the same was true for NADH compared with the *Kcat* of NAD+. These results indicated that the efficiency of recombinant LDH-catalyzed pyruvate reduction reaction was higher than that of the lactate reaction. In the presence of the coenzyme APAD, rLDH catalyzed the lactate oxidation reaction at a much higher rate than that in the presence of NAD+. Actually, the *Km* values of LDH varied significantly among different species [33,34,35,36], which suggested that the differences in the amino acid sequence and spatial structure of LDH among different species may be functionally reflected in the ability to adapt to the parasitic environment, substrate affinity or enzymatic properties such as catalytic efficiency. These differences can be used as important reference values for the physiological and biochemical studies of parasites, as well as in immunodiagnosis and drug development [37]. In the reduction and oxidation reactions, the inhibition efficiency of Gsp was much higher than that of Thio, Niacin and Oxalate. Gsp is a polyphenol dinaphthalene asymmetric terpene extracted from cotton seeds, which has a wide range of biological activities. It can inhibit *Tg*LDH [33], *Sj*LDH [35], and *Clonorchis sinensis* LDH [36]. The epitopes 104 aa-111 aa located on the surface of LDH are important binding sites of NAD and substrate pyruvate. Key catalytic sites of 112 R, 172 D and 199 H formed the catalytic center adjacent to the epitopes. Most of binding sites of NAD and substrate pyruvate are located in this region, and others are concentrated around this region. The binding of corresponding antibodies in the region not only mediates immune attack, but also acts as an enzyme-specific inhibitor, preventing the binding of substrate pyruvate and causing the accumulation of pyruvate in cells to cause the death of the parasite [38]. Therefore, we evaluated in vitro how antibodies affect the enzymatic activity of r*Sm*LDH. Our results showed that the anti-*Sm*LDH antibody produced a mild and dose-dependent inhibition of rS*m*LDH enzyme activity. In the future, we hope to conduct more in-depth studies on the function of *Sm*LDH to explore the important role of *Sm*LDH in the growth and development of the neglected parasite.

## 5. Conclusions

In this study, 19 novel LDH members in *S. mansoni* were identified. A phylogenetic analysis of LDHs from cestode and trematode species showed a significant amount of variability. Meanwhile, we successfully cloned, produced, and investigated the enzymatic properties of the *Sm*LDH recombinant protein. Purified r*Sm*LDH showed high activity at pH 4.5 and an optimum temperature of 37 °C in the reduction reaction. In the oxidation reaction, optimal values for pH and temperature were 9.0 and 30 °C, respectively. Gossypol was displayed as a powerful enzyme inhibitor in both reduction and oxidation reactions. Our study suggests that inhibiting the activity of *Sm*LDH probably affects the growth and development of *S. mansoni*. Additionally, LDH may be a good diagnostic molecule and potential target for anti-tapeworm drugs.

## Figures and Tables

**Table 1:** Annotation characteristics of lactate dehydrogenase in *Spirometra mansoni*.

Sequences	Gene ID	LDH Domain Coordinates	Domain Length (aa)	Protein Length (aa)
*Sm*LDH1	ADK62519.1	23–162, 165–333	139,168	338
*Sm*LDH2	SPER_0001136701	23–162, 165–332	139,167	334
*Sm*LDH3	SPER_0001938001	23–162, 165–287	139,122	289
*Sm*LDH4	SPER_0002127101	14–86	72	140
*Sm*LDH5	SPER_0002457401	1–92	91	93
*Sm*LDH6	SPER_0002847401	17–78, 97–153	61,56	156
*Sm*LDH7	TRINITY_DN30655_c0_g1_i1	24–163, 166–334	139,168	335
*Sm*LDH8	TRINITY_DN32812_c0_g1_i11	3–89, 92–150	86,58	152
*Sm*LDH9	TRINITY_DN32812_c0_g1_i9	3–89, 92–259	86,167	261
*Sm*LDH10	TRINITY_DN32812_c0_g1_i8	92–231, 234–353	139,119	358
*Sm*LDH11	TRINITY_DN32812_c0_g1_i4	11–96	85	97
*Sm*LDH12	TRINITY_DN32812_c0_g1_i7	26–122	96	123
*Sm*LDH13	TRINITY_DN32812_c0_g1_i6	26–69, 72–192	43,120	196
*Sm*LDH14	TRINITY_DN32812_c0_g1_i1	21–147	126	148
*Sm*LDH15	TRINITY_DN32812_c0_g1_i10	1–105, 108–275	104,167	277
*Sm*LDH16	TRINITY_DN32381_c0_g2_i1	7–50, 53–221	43,168	222
*Sm*LDH17	TRINITY_DN32381_c0_g2_i2	56–195, 198–366	139,168	367
*Sm*LDH18	TRINITY_DN29226_c0_g1_i1	2–88, 91–265	86,174	266
*Sm*LDH19	TRINITY_DN29226_c0_g1_i4	41–180, 183–357	139,174	358

## Data Availability

The data supporting the conclusions of this article are included within the article.

## Supplementary Materials

The following supporting information can be downloaded at https://www.mdpi.com/article/10.3390/ani13233642/s1. Figure S1: *Sm*LDH functional prediction results; Figure S2: Effect of pH and temperature on rLDH enzyme activity; Figure S3: Results of logarithmic values of inhibitor concentrations versus residual enzyme activity; Figure S4: Results of anti-rLDH antibody concentration logarithm and residual enzyme activity; Table S1: Primers used in qRT-PCR analysis; Table S2: Summary of LDHs in platyhelminthes.
