# Supplementary material for: Lactate Dehydrogenase Gene Family in Spirometra mansoni (Cestoda: Diphyllobothriidea)—Phylogenetic Patterns and Molecular Characteristics
Source: Animals (Basel). 2023 Nov 24;13(23):3642. doi: 10.3390/ani13233642 (PMC10705530; doi:10.3390/ani13233642)
Supplement: Supplementary file 1 [file animals-13-03642-s001.zip › animals-2659514-supplementary.pdf]

SUPPORTING INFORMATION

Lactate dehydrogenase gene family in *Spirometra mansoni* (Cestoda: Diphyllobothriidea) — phylogenetic patterns and molecular characteristics

Table S1. Primers used in qRT-PCR analysis.

Table S2. Summary of LDHs in platyhelminthes.

Fig S1. *Sm*LDH functional prediction results.

Fig S2. Effect of pH and temperature on rLDH enzyme activity.

Fig S3. Results of logarithmic values of inhibitor concentrations versus residual enzyme activity.

Fig S4. Results of anti-rLDH antibody concentration logarithm and residual enzyme activity.

Table S1. Primers used in qRT-PCR analysis.

Gene	Primer name	Sequence (5'-3')	Product size (bp)
SmLDH1	SmLDH1-S<br>SmLDH1-A	CGATGTTATGGTTGACAAAGTG<br>AATCTAAAACGGGCGGAAT	363
SmLDH2	SmLDH2-S<br>SmLDH2-A	GAAAGAACTCGCTAATGAA<br>TTGCTGACCCTCTTGC	88
SmLDH3	SmLDH3-S<br>SmLDH3-A	GATGAAAGAACTCGCTAATG<br>GCAAGTTGTGGGATGAGTT	258
SmLDH4	SmLDH4-S<br>SmLDH4-A	CGGTCTGGAGCAAGGT<br>TCATCATCCTGTCCCATT	73
SmLDH5	SmLDH5-S<br>SmLDH5-A	CGCTTGGACTGGCTACA<br>GTCGCTGAGTTGTTGAAAGAGGAT	179
SmLDH6	SmLDH6-S<br>SmLDH6-A	AGTTATCGGAGCAGGGTC<br>TCACCAGCAACCCTTT	111
SmLDH7	SmLDH7-S<br>SmLDH7-A	CACCGTCAGTAGTTTCGC<br>TCGGAGCAGAAGGAATCAGTCAA	105
SmLDH8	SmLDH8-S<br>SmLDH8-A	AGATTCGCCTTCCCTTTG<br>GTGGCTTTCTATCCTGTCATT	175
SmLDH9	SmLDH9-S<br>SmLDH9-A	AAATGACAGGATAGAAAGCCACAA<br>CTGCCAGTGTCAACGAAC	248
SmLDH10	SmLDH10-S<br>SmLDH10-A	GAGAAAGGCGTGTCCG<br>TGTGAAACCTGCGAAC	162
SmLDH11	SmLDH11-S<br>SmLDH11-A	GAGTTTGGGATAGGAAGT<br>ATGCGTCTTGCTTACAATGGAG	154
SmLDH12	SmLDH12-S<br>SmLDH12-A	AAATGACAGGATAGAAAGCCACAA<br>CTGCCAGTGTCAACGAAC	248
SmLDH13	SmLDH13-S<br>SmLDH13-A	GCCATACTTCCACTGCG<br>GGGCGAATCTCGTCTGG	255
SmLDH14	SmLDH14-S<br>SmLDH14-A	ATCCAGACGAGATTCGCCTTCC<br>GTGGCTTTCTATCCTGTCATTTCTG	140
SmLDH15	SmLDH15-S<br>SmLDH15-A	AGATTCGCCCTCCCTTTG<br>GTGGCTTTCTATCCTGTCATTT	198
SmLDH16	SmLDH16-S<br>SmLDH16-A	TGAGAAACGCACAGGTC<br>TACGCTTCTCCATTCTGT	165
SmLDH17	SmLDH17-S<br>SmLDH17-A	GCTCTTTGACTGACCCTC<br>CTACGCTTCTCCATTCTGT	129
SmLDH18	SmLDH18-S<br>SmLDH18-A	GCCCTTCAGTTTACCTTC<br>GCTTGGACTGGCTACAT	103
SmLDH19	SmLDH19-S<br>SmLDH19-A	TGTAGCCAGTCCAAGCG<br>TCCTATCACGACCTGTTG	365
GAPDH	GAPDH-S<br>GAPDH-A	AGCAACCTCGTTGATGTCGT<br>TGAATTGACCGTGGGTGGAG	97

Table S2. Summary of LDHs in other platyhelminthes

Class	Family	Genus	SPECIES	SS	SEQUENCE ID
Cestoda	Diphyllobothriidea	*Dibothriocephalus*	*D. latus*	5	DILT_0000946301, DILT_0000453201, DILT_0000465001, DILT_0000587101, DILT_0000278001
		Spirometra	*S. erinaceieuropaei*	19	SmLDH1_ADK62519, SmLDH2_SPER0001136701, SmLDH3_SPER0001938001, SmLDH4_SPER0002127101, SmLDH5_SPER0002457401, SmLDH6_SPER0002847401, SmLDH7_DN30655_c0_g1_i1, SmLDH8_DN32812_c0_g1_i11, SmLDH9_DN32812_c0_g1_i9, SmLDH10_DN32812_c0_g1_i8, SmLDH11_DN32812_c0_g1_i4, SmLDH12_DN32812_c0_g1_i7, SmLDH13_DN32812_c0_g1_i6, SmLDH14_TRINITY_DN32812_c0_g1_i1, SmLDH15_DN32812_c0_g1_i10, SmLDH16_DN32381_c0_g2_i1, SmLDH17_DN32381_c0_g2_i2, SmLDH18_DN29226_c0_g1_i1, SmLDH19_DN29226_c0_g1_i4
		Schistocephalus	*S. solidus*	5	SSLN_0001553101, SSLN_0001884001, SSLN_0000665501, SSLN_0002048901, SSLN_0001267001
	Taeniidae	*Echinococcus*	*E. canadensis*	7	EcG7_08021, EcG7_04628, EcG7_00202, EcG7_08839, EcG7_07350, EcG7_09441, EcG7_06430
			E. granulosus	23	EGR_03412, EGR_11159, EGR_02865, EGR_01010, EGR_07010 EgrG_000660800, EgrG_000467700, EgrG_001185100, EgrG_001185000, EgrG_000608500, EgrG_000634800, EgrG_000661000, ADK25713, AFA35122, CDS18772, CDS18770, CDS18561, CDS18320, CDS19376, XP_024345180, XP_024353608, XP_024353062, XP_024355078

			E. multilocularis	14	EmuJ_000467700, EmuJ_000007100, EmuJ_001185000, EmuJ_000608500, EmuJ_000661000, EmuJ_000660800, EmuJ_001185100, EmuJ_000634800, CUT99511, CUT99509, CUT99280, CUT99024, CDS37431, CDI96497
		Taenia	*T. asiatica*	15	TASs00006g01735, TASs00004g01136, TASs00143g08337, TASs00010g02429, TASs00022g03629, TASs00010g02428, TASs00261g09518, TASs00045g05221, TASK_0000370001, TASK_0000488101, TASK_0000036701, TASK_0000266701, TASK_0000166101, TASK_0000897001, TASK_0000332901
			T. multiceps	6	Tm1G001697, Tm3G008999, Tm3G008538, Tm3G008801, Tm1G001282, Tm1G001281
			T. saginata	7	TSAs00003g01040, TSAs00063g07180, TSAs00011g02700, TSAs00269g10395, TSAs00028g04543, TSAs00026g04348, TSAs00026g04349
			T. solium	8	TsM_000436000, TsM_000048200, TsM_000460300, TsM_000807200, TsM_000798800, TsM_000566100, ADV35657, ADV35656
		Hydatigera	*H. taeniaeformis*	4	TTAC_0000780101, TTAC_0000880801, TTAC_0000672301, TTAC_0000614401
	Hymenolepididae	*Hymenolepis*	*H. diminuta*	14	WMSIL1_LOCUS8692, WMSIL1_LOCUS8449, WMSIL1_LOCUS6753, WMSIL1_LOCUS6544.1, WMSIL1_LOCUS6544.2, WMSIL1_LOCUS644.1, WMSIL1_LOCUS644.2 ,WMSIL1_LOCUS4114 HDID_0000660801, HDID_0000561501, HDID_0000060301, HDID_0000060201, HDID_0000534801, HDID_0000846001
			H. nana	5	HNAJ_0000388801, HNAJ_0000045201, HNAJ_0001035001, HNAJ_0000149201, HNAJ_0000045301

			H. microstoma	6	HmN_000705800,HmN_000478500,HmN_000063100, HmN_000063000, CDS26883, CDS32958
	Mesocestoididae	*Mesocestoides*	*M. corti*	11	MCU_010028-RA, MCU_014229-RA, MCOS_0001007601, MCU_006190-RA, MCU_006567-RB, MCU_006567-RC, MCU_008646-RA, MCU_011195-RA, MCU_011195-RB, MCU_010950-RA ,MCU_003955-RB
Trematoda	Schistosomatidae	*Schistosoma*	*S. japonicum*	29	EWB00_010981.1, EWB00_010981.2, EWB00_000090.1, EWB00_000090.2, EWB00_000090.3, EWB00_000090.4, EWB00_000090.5, EWB00_000090.6, EWB00_008117, EWB00_010161.1, EWB00_010161.2, EWB00_010161.3, EWB00_010161.4, EWB00_008117.1, EWB00_008117.2, EWB00_008117.3, EWB00_003119, Sjp_0054470, Sjp_0041910, Sjp_0024400, Sjp_0054460, Sjp_0110090 ACM17843.1, TNN13174.1, TNN04890.1, TNN04889.1, AAP06144.1, AAO59420.2 ,CAX70608.1
			S. haematobium	6	MS3_0020905.1, MS3_0013775.1, MS3_0018505.1, MS3_0016103.1, MS3_0020713.1, XP_012801751.2
			S. mansoni	8	Smp_033040.1, Smp_035270.1, Smp_047370.1, Smp_038950.1, Smp_070860.1, Smp_070860.2, Smp_038960.1, Smp_038960.2
			S. margrebowiei	6	SMRZ_0002353501, SMRZ_0000009301, SMRZ_0000280301, SMRZ_0000448701, SMRZ_0002427601, SMRZ_0001892101
			S. mattheei	3	SMTD_0002071001, SMTD_0001380401, SMTD_0000184401
			S. rodhaini	6	SROB_0002247801, SROB_0001485701, SROB_0000648401, SROB_0001979101, SROB_0002058901, SROB_0000334801

			S. bovis	9	DC041_0007377, DC041_0008291, DC041_0002698, DC041_0000949, DC041_0012974, DC041_0012344, RTG90372.1, RTG80559.1, RTG80558.1
			S. curassoni	7	SCUD_0000539301, SCUD_0001337701, SCUD_0001603901, SCUD_0002203501, SCUD_0000531801, SCUD_0001533401, SCUD_0000553001
	Opisthorchiidae	*Clonorchis*	*C. sinensis*	17	csin112591, csin110424, csin110966, csin110969, csin107685, CSKR_111940, CSKR_103326, CSKR_108044, CSKR_107406, CSKR_108045, CSKR_108047, AAV80238.1, KAG5444587.1, KAG5444585.1, KAG5442918.1, GAA52251.1, ABZ82030.1
	Fasciolidae	*Fasciolopsis*	*F. buski*	4	FBUS_08099, FBUS_02962, FBUS_06581, KAA0185729.1
		Fasciola	*F. gigantica*	8	FGIG_06624, FGIG_07754, FGIG_11029, FGIG_09125, FGIG_08365, FGIG_05200, FGIG_12400, TPP62930.1
			F. hepatica	15	D915_006510, D915_003689, D915_003688, D915_004472, D915_004345, D915_010544, D915_009078, maker-scaffold10x_158_0.11, maker-scaffold10x_158_0.0, maker-scaffold10x_120, maker-scaffold10x_7, maker-scaffold10x_377, THD25592.1, THD24834.1, THD22890.1
	Paragonimidae	*Paragonimus*	*P. westermani*	5	DEA37_0003442, DEA37_0011538, DEA37_0012094, DEA37_0010569, KAA3681067.1
			P. heterotremus	1	KAF5398688.1

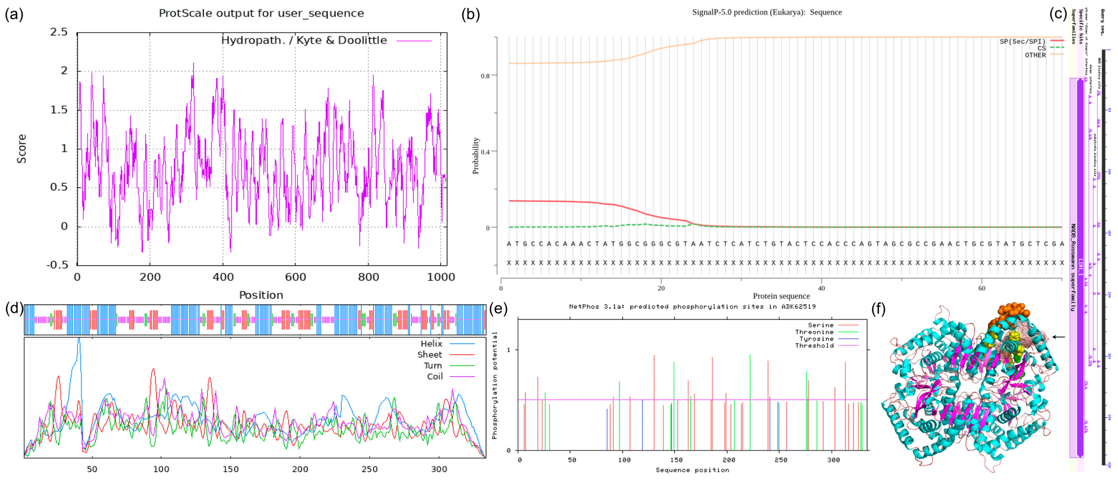

Fig. S1. *Sm*LDH functional prediction results. (a) affinity prediction; (b) signal peptide prediction; (c) functional structure domain prediction; (d) structure prediction; (e) phosphorylation site prediction; (f) 3D structure prediction.

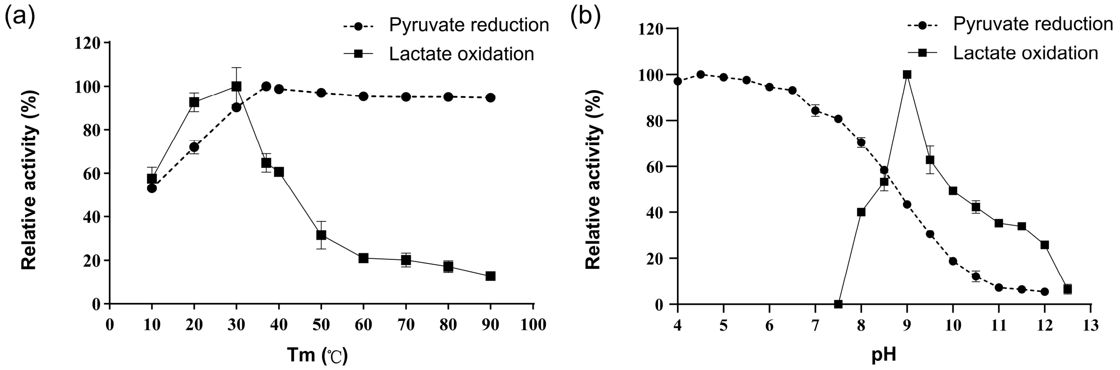

Fig. S2. Effect of pH and temperature on rLDH enzyme activity. (a) Effect of temperature; (b) Effect of pH.

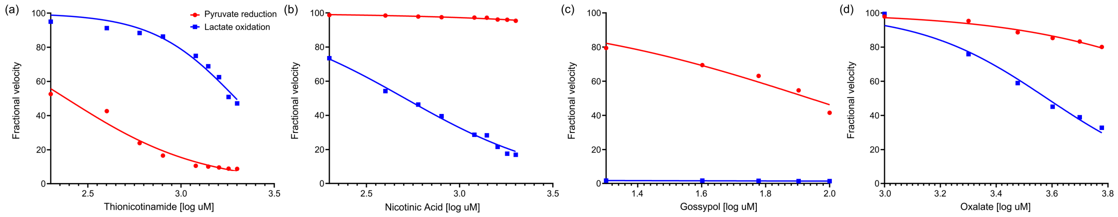

Fig. S3. Results of logarithmic values of inhibitor concentrations versus residual enzyme activity. (a) Thio inhibitor; (b) Niacin inhibitor; (c) Gsp inhibitor; (d) Oxalate inhibitor.

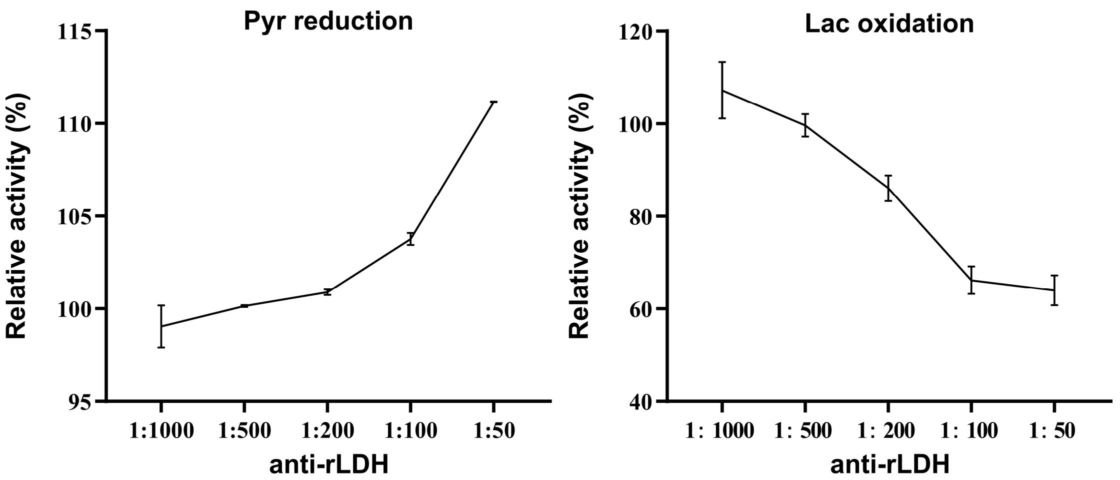

Fig. S4. Results of anti-rLDH antibody concentration logarithm and residual enzyme activity.
